# HIV therapy in an ageing population — the challenge of polypharmacy

**DOI:** 10.1186/1758-2652-13-S4-O26

**Published:** 2010-11-08

**Authors:** DJ Back

**Affiliations:** 1 Pharmacology Research Laboratories, University of Liverpool, Liverpool, UK

## 

Pharmacokinetic drug interaction studies performed during the drug development process, or post-licensing provide the substantive data base from which recommendations regarding the use of certain drug combinations are made. However given the sheer number of potential interactions, especially in the context of an ageing population with inevitable co-morbidities and polypharmacy, we need to be able to make informed decisions even in the absence of study data. Thus knowledge of drug handling (the role of various metabolic enzymes and transporters etc.) is essential so that pre-clinical data (determining whether the drug is a substrate or an inhibitor of a particular enzyme or transporter; use of in vitro/in vivo extrapolation tools etc.) can be the basis for deciding how to proceed. However we also need to appreciate that with the ageing process drug absorption and clearance can alter so the challenge then is to understand how this may impact on the magnitude of a drug-drug interaction. While the major focus in the HIV field has been on CYP450 enzymes (for the obvious reason that many of the drugs are extensively metabolised and/or are inducers/inhibitors) there is a growing awareness of the key role for other proteins - in particular UDP-glucuronyltransferases (UGTs) and transporters (ABC transporters such as P-gp, MRPs; SLCO transporters such as OATP1B1, OCTs, OATs). This is a rapidly emerging field and one which is going to impact on our understanding of mechanisms of drug-drug interactions. In addition the variable expression of enzymes and transporters due to pharmacogenetic changes is another important consideration. Unexpected interactions will continue to emerge and will need to be managed. Ultimately the key to management of patients on multiple drugs is clinical vigilance, access to adequate resources to help inform (e.g. web based resources), and in some cases the careful use of therapeutic drug monitoring.

